# Auditory Psycholinguistic Abilities and Cognitive Performance in Children with Developmental Language Disorder

**DOI:** 10.3390/bs16071199

**Published:** 2026-07-16

**Authors:** Beatriz María Bonillo-Llavero, Alejandro Cano-Villagrasa, Miguel López-Zamora

**Affiliations:** 1 UCAM Universidad Católica de Murcia, 30107 Murcia, Spain; 2 Facultad de Ciencias de la Salud, Universidad Internacional de Valencia, 46002 Valencia, Spain; 3 Departamento de Psicología, Facultad de Ciencias de la Salud, Universidad Europea de Canarias, 38300 La Orotava, Spain; 4 Departamento de Psicología Evolutiva y de la Educación, Universidad de Málaga, 29010 Málaga, Spain

**Keywords:** developmental language disorder, auditory psycholinguistic abilities, cognitive performance, working memory, fluid reasoning, processing speed

## Abstract

Developmental Language Disorder (DLD) is characterized by persistent difficulties in language acquisition and use and is frequently accompanied by variability in cognitive performance. This study compared auditory psycholinguistic abilities and selected cognitive outcomes in children with DLD and typically developing (TD) peers and examined whether associations between these domains were robust within each group. Eighty Spanish-speaking children aged 7–9 years participated (40 DLD, 40 TD). Four auditory subtests of the Illinois Test of Psycholinguistic Abilities assessed auditory comprehension, auditory association, auditory integration, and auditory sequential memory. Age-scaled scores from selected WISC-V subtests represented verbal comprehension, working memory, fluid reasoning, and processing speed. Between-group differences were examined with independent-samples *t* tests and sensitivity analyses controlling for paternal education. Pearson correlations were supplemented by Spearman correlations and false-discovery-rate corrections. Children with DLD scored lower than TD peers on all auditory psycholinguistic and cognitive measures (Cohen’s d = 1.35–2.55). The between-group differences remained significant after accounting for paternal education; for auditory integration, significant differences were observed at both levels of paternal education despite a significant interaction. Three Pearson correlations reached nominal significance before correction, but none was replicated as statistically significant by Spearman analysis or survived correction for multiple testing. The findings therefore indicate broad between-group differences but limited evidence for stable within-group coupling between the auditory psycholinguistic and cognitive measures assessed. Comprehensive assessment should consider language-related and cognitive domains separately rather than assuming a single explanatory mechanism.

## 1. Introduction

Developmental Language Disorder (DLD) is a prevalent neurodevelopmental condition, characterized by persistent difficulties in the acquisition and use of language in the absence of intellectual disability, uncorrected hearing loss, or known neurological injury. It affects approximately 7–8% of preschool-aged children, underscoring its public health impact and the need for greater mechanistic clarity (Tomblin et al., 1997). The CATALISE consensus recommendations have emphasized function-based identification and clarified that a low nonverbal IQ (NVIQ) is not a requirement for diagnosis, improving comparability across studies and clinical decision-making (Bishop et al., 2016). Accordingly, cognitive thresholds used in individual studies should be understood as sampling decisions rather than as universal diagnostic criteria. Complementary population-based research shows that imposing NVIQ thresholds can alter prevalence estimates and case composition—an analytic artifact that highlights how definitional choices influence inference (Norbury et al., 2016). Beyond prevalence, children with DLD face an elevated risk of long-term academic and psychosocial challenges, including literacy difficulties, low educational attainment, and reduced quality of life, with effects persisting into adolescence and adulthood (Conti-Ramsden et al., 2018).

In the DLD literature, it is essential to establish a clear separation of constructs. Auditory psycholinguistic abilities are higher-level operations that map acoustic input to lexico-semantic and structural representations. In this work, we operationalize four core psycholinguistic abilities: auditory comprehension, auditory association, auditory integration, and auditory sequential memory. This distinction is crucial: whereas evidence of atypical speech perception in DLD documents lower-level perceptual phenomena—e.g., reduced sensitivity to stress/rhythm, impaired identification of phonetic features in quiet and in noise (Richards & Goswami, 2015; Ziegler et al., 2005, 2011)—psycholinguistic abilities index the efficiency with which encoded signals are organized and exploited for meaning and structure at a cognitive–linguistic level.

Converging findings associate DLD with limitations in verbal working memory and short-term memory, with direct consequences for online comprehension (Montgomery, 2000, 2003). Systematic reviews consistently replicate these deficits (Archibald & Gathercole, 2006), and a meta-analysis demonstrates moderate-to-large group differences in visuospatial working memory, indicating that limitations extend beyond phonology to domain-general capacity (Vugs et al., 2013). Differences in executive function persist after adjusting for age and NVIQ, encompassing working memory with executive load, verbal fluency, inhibition, and planning, implicating control processes that undergird goal-directed language behavior (Henry et al., 2012). In parallel, a robust literature documents a generalized slowing of processing speed in DLD across linguistic and non-linguistic tasks (Miller et al., 2001). Joint models suggest tight coupling among speed, phonological short-term memory (STM), and language outcomes (Leonard et al., 2007; Montgomery & Windsor, 2007). Taken together, these data motivate a framework in which psycholinguistic abilities interact with domain-general resources (capacity, speed, and control) to support language performance.

Empirically, each of these cognitive domains is plausibly linked to psycholinguistic operations. Verbal comprehension integrates lexico-semantic and structural information; compromised psycholinguistic input (e.g., weaker auditory comprehension or association) may limit the building blocks available for propositional integration, particularly under time pressure. Working memory supports the maintenance and manipulation of partial analyses and referential dependencies; deficits in auditory sequential memory and integration may reduce the fidelity of maintained units, increasing the risk of decay or interference (Archibald & Gathercole, 2006; Montgomery, 2003). Processing speed reflects the rate at which representations are retrieved and bound; generalized slowing (Miller et al., 2001) could jointly depress the efficiency with which partial cues are combined. Finally, fluid reasoning involves relational abstraction and analogical mapping; psycholinguistic comprehension and association may serve as language-proximal scaffolds for discovering and maintaining relational structure, leading to a grounded pathway from language-specific abilities to broader cognitive performance (Gillam et al., 2019).

Longitudinal and outcome-oriented studies reinforce these links. Differential language growth trajectories in DLD are predicted by early profiles of working memory and processing speed, with cumulative consequences for literacy and academic achievement (Conti-Ramsden et al., 2018; Leonard et al., 2007; Montgomery & Windsor, 2007). Notably, reducing temporal demands (e.g., with a slower input rate or extended processing time) can partially ameliorate comprehension difficulties in DLD (Montgomery, 2005, 2006), consistent with a capacity/efficiency interpretation.

Despite extensive evidence of language and cognitive difficulties in DLD, three gaps remain. First, comparatively few studies have examined several higher-level auditory psycholinguistic abilities alongside distinct cognitive outcomes within the same design. Second, associations observed in pooled samples may reflect large mean differences between diagnostic groups rather than covariance within each group. Third, evidence from Spanish-speaking school-age samples using standardized Spanish-language measures remains limited. Addressing these gaps requires a design that combines direct group comparisons with separate within-group association analyses and robustness checks for distributional assumptions and multiple testing.

Accordingly, the present study examined auditory comprehension, auditory association, auditory integration, and auditory sequential memory in relation to verbal comprehension, working memory, fluid reasoning, and processing speed in children with DLD and TD peers. The primary objective was to estimate between-group differences. The secondary objective was to explore within-group associations using both Pearson and Spearman coefficients, with confidence intervals and correction for multiple testing.

We hypothesized that (H1) children with DLD would obtain lower scores than TD peers on all four auditory psycholinguistic measures and all four cognitive measures. We further expected (H2) selective positive within-group associations, particularly between auditory sequential memory and working memory and between auditory comprehension or association and verbally mediated or relational reasoning outcomes. Because prior evidence is heterogeneous and the sample was modest, H2 was treated as exploratory and evaluated with parametric and non-parametric correlations and multiplicity correction.

## 2. Materials and Methods

### 2.1. Study Design

This study used a cross-sectional, comparative, and correlational design. The analyses addressed three objectives: (a) to compare children with DLD and TD peers on auditory psycholinguistic abilities and cognitive performance; (b) to examine within-group associations between the two domains; and (c) to evaluate the robustness of the results through covariate-adjusted group comparisons and non-parametric correlation analyses. The sample size was determined by the number of eligible participants available during the recruitment period rather than by an a priori power analysis. A post hoc sensitivity analysis was therefore conducted using the power-analysis functions implemented in statsmodels to characterize the magnitude of effects that could be detected with the available sample. With 40 participants per group, a two-tailed significance level of α = 0.05, and 80% statistical power, the minimum detectable standardized mean difference for an independent-samples comparison was approximately *d* = 0.63. For the within-group correlation analyses, with *n* = 40, two-tailed α = 0.05, and 80% power, the minimum detectable correlation was approximately |*r*| = 0.43. Consequently, the study had adequate sensitivity for large between-group differences but limited sensitivity for small-to-moderate within-group associations. The correlation analyses were therefore considered exploratory.

### 2.2. Participants

The analytic sample comprised 80 Spanish-speaking children aged 7–9 years: 40 children with DLD and 40 TD peers. The total sample had a mean age of 8.08 years (SD = 0.31; range = 7.3–8.9). Children in the DLD group had a prior clinical or educational diagnosis documented in school or clinical records and confirmed by parents or legal guardians at enrolment. Although some source records used the historical label specific language impairment (SLI), the term DLD is used throughout this manuscript in accordance with contemporary terminology. TD children had no reported history of language, learning, neurological, sensory, or neurodevelopmental disorders according to parent reports and school records.

Inclusion criteria for all participants were: (a) age between 7 and 9 years; (b) Spanish as the language of schooling and assessment; (c) normal or corrected-to-normal vision and hearing; and (d) documentation of general intellectual functioning within the typical range, operationalized as Full Scale IQ (FSIQ) ≥ 85. FSIQ was used only as an eligibility criterion and was not included as an analytic outcome. This threshold was used to delimit the analytic sample and to reduce the potential influence of global intellectual disability on the between-group comparisons. It should not be interpreted as a general diagnostic requirement for DLD, in line with current consensus recommendations indicating that low nonverbal ability alone does not preclude a diagnosis of DLD. Exclusion criteria were uncorrected hearing or visual impairment, known neurological injury, autism spectrum disorder, attention-deficit/hyperactivity disorder, intellectual disability, or educational circumstances likely to interfere substantially with language development or assessment validity, such as prolonged non-standard instruction, chronic absenteeism, late full-immersion second-language schooling, or enrolment in specialized settings for conditions other than DLD.

**Figure 1:**
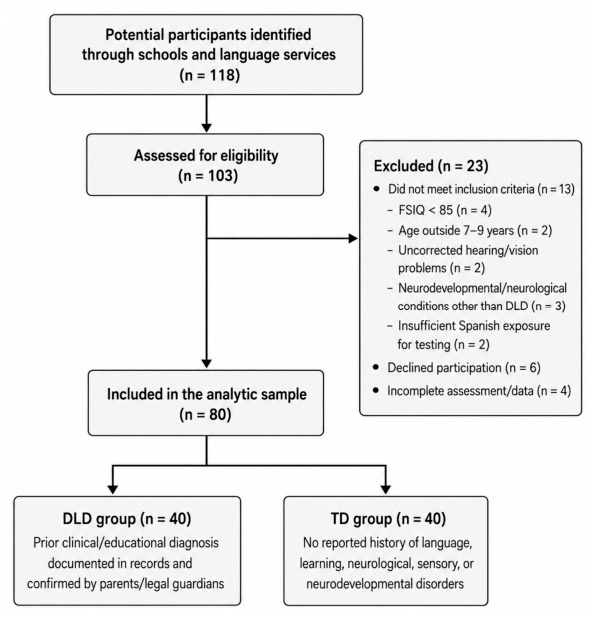
Participant flowchart.

Sociodemographic information included age, sex, socioeconomic level, paternal and maternal education, family structure, and school type. Socioeconomic level and the FSIQ eligibility criterion were invariant in the analytic dataset and were reported descriptively. Paternal education differed between groups and was therefore included as a covariate in sensitivity analyses of the eight primary group comparisons. The participant recruitment and selection process is summarized in Figure 1.

### 2.3. Instruments and Measures

#### 2.3.1. Auditory Psycholinguistic Abilities

Auditory psycholinguistic abilities were assessed using the Spanish adaptation of the Illinois Test of Psycholinguistic Abilities (ITPA; Ballesteros & Cordero, 2004), applying the age-based Spanish normative data provided in the manual. The Spanish manual reports internal-consistency estimates based on Kuder–Richardson and Hoyt coefficients. Across the ITPA subtests, reliability coefficients generally range from 0.70 to 0.94, whereas the global psycholinguistic quotient shows higher reliability estimates, ranging from 0.95 to 0.99. These values indicate adequate-to-high reliability for the individual subtests and very high reliability for the global score. However, because the present study analysed four individual subtests rather than the global quotient, the psychometric interpretation of the study variables should be based primarily on the reliability of the corresponding subtests.

Four auditory subtests were selected because they represented the main language-processing abilities examined in the study: auditory comprehension, auditory association, auditory integration, and auditory sequential memory. Auditory comprehension assesses the ability to derive meaning from spoken language and process lexical-semantic and syntactic information. Auditory association assesses the ability to establish semantic and functional relationships between verbal stimuli. Auditory integration evaluates the reconstruction or completion of partially presented verbal material using linguistic and contextual knowledge. Auditory sequential memory assesses the short-term retention and serial reproduction of auditorily presented verbal information.

Subtests were administered individually under standardized conditions. Raw scores were converted according to age-appropriate Spanish norms and treated as continuous scores. Higher scores indicated stronger auditory psycholinguistic performance. These four variables were examined as outcomes in the between-group comparisons and as correlates of the selected cognitive measures in the within-group analyses.

#### 2.3.2. Cognitive Outcomes

Cognitive performance was assessed using selected subtests from the Wechsler Intelligence Scale for Children–Fifth Edition (WISC-V; Wechsler, 2014). Verbal comprehension was represented by the Similarities subtest, working memory by the Digit Span subtest, fluid reasoning by the Matrix Reasoning subtest, and processing speed by the Coding subtest. Age-scaled subtest scores were analysed rather than full composite index scores. Higher scores indicated better performance. Accordingly, the findings should be interpreted as performance on the selected tasks and not as estimates of the complete WISC-V indices or global intellectual functioning.

Verbal comprehension was used to represent verbally mediated reasoning and lexical-conceptual knowledge; working memory reflected the short-term maintenance and manipulation of information; fluid reasoning represented relational and nonverbal reasoning; and processing speed indexed efficiency in rapid visual scanning, coding, and graphomotor output under time constraints. FSIQ was not analysed as an outcome because it was used solely as an eligibility criterion.

#### 2.3.3. Sociodemographic and Eligibility Variables

Age was recorded in years. Sex was coded as boy or girl. Socioeconomic level was classified from parent-reported information and school records. Paternal and maternal education were dichotomized for analysis as university versus non-university education to allow stable group comparisons. Family structure was coded as nuclear versus single-parent or other family structures, and school type was coded as public versus private/concerted. These variables were used to describe the sample and evaluate group comparability before conducting the main analyses.

### 2.4. Procedure

Participants were recruited through educational centres and specialized language services. Families received written and oral information about the study aims, assessment procedures, confidentiality safeguards, and the voluntary nature of participation. Written informed consent was obtained from parents or legal guardians, and verbal assent was obtained from the children before assessment. For children in the DLD group, diagnostic status and eligibility criteria were verified using school or clinical documentation and parent confirmation. For TD children, the absence of developmental, sensory, neurological, or learning concerns was verified through parent reports and school records.

Assessments were conducted individually in a quiet room by the same examiner, a qualified speech-language therapist and psychologist with experience in child language and cognitive assessment. The examiner did not participate in the original diagnostic classification; however, complete blinding to group status could not be guaranteed because diagnostic information could be apparent from the records and the children’s profiles. As a single examiner administered and scored all measures, inter-rater reliability was not applicable. Testing was divided into two sessions of approximately 45 min to reduce fatigue. ITPA measures were administered in the first session and WISC-V measures in the second. Short breaks were offered when needed, and all records were checked, anonymized, and entered into a secure database.

### 2.5. Data Preparation and Statistical Analysis

Analyses were conducted in Python 3.13.5 using SciPy 1.17.0, statsmodels 0.14.6, pandas 2.2.3, NumPy 2.3.5, and Matplotlib 3.10.8. False-discovery-rate correction was performed using the Benjamini–Hochberg procedure implemented in statsmodels. Bootstrap confidence intervals were estimated using custom resampling procedures with a fixed random seed of 2026 to ensure reproducibility. All statistical tests were two-tailed, with α = 0.05. The dataset was screened for missing values, coding errors, extreme outliers, and distributional anomalies. Within-group distributions were examined using descriptive indices, histograms, Q–Q plots, and Shapiro–Wilk tests. Equality of variance for the between-group comparisons was assessed using Levene’s test. Given the discrete nature of the scaled scores and the observed departures from normality, Pearson correlations were supplemented with Spearman rank-order correlations as a non-parametric sensitivity analysis. Equal-variance independent-samples *t* tests were retained for the primary group comparisons because the groups were equal in size, no extreme outliers were identified, and the homogeneity-of-variance assumption was satisfied.

Descriptive statistics were calculated for sociodemographic, auditory psycholinguistic, and cognitive variables. Group comparability was assessed using an independent-samples *t* test for age and chi-square tests for categorical variables. Variables without between-participant variability were reported descriptively and were not tested inferentially.

Independent-samples *t* tests were used to compare the DLD and TD groups on the four auditory psycholinguistic measures and the four cognitive measures. Mean differences were calculated as DLD minus TD, so negative values indicated lower performance in the DLD group. Cohen’s *d* was calculated using the pooled standard deviation and reported in the TD-minus-DLD direction, so positive values indicated higher scores in the TD group. Ninety-five percent confidence intervals were calculated for the mean differences, and 95% bootstrap confidence intervals for Cohen’s *d* were based on 5000 resamples. Benjamini–Hochberg false-discovery-rate correction was also applied across the eight primary between-group comparisons.

Because paternal education differed between groups, each primary outcome was additionally examined using a linear model including group status and paternal university education. Before interpreting the adjusted models, the homogeneity-of-regression-slopes assumption was assessed by testing the group-by-paternal-education interaction for each outcome. When the interaction was non-significant, a reduced model containing the main effects of group and paternal education was estimated. When the interaction was statistically significant, simple group effects were estimated separately at each level of paternal education rather than reporting a single common adjusted group effect.

Pearson correlations were calculated separately within the DLD and TD groups for each pairing of an auditory psycholinguistic measure with a cognitive measure. Ninety-five percent confidence intervals for Pearson coefficients were obtained using Fisher’s *z* transformation. Spearman’s ρ was calculated as a non-parametric sensitivity analysis, with 95% bootstrap confidence intervals based on 2000 resamples. Benjamini–Hochberg false-discovery-rate correction was applied separately to the 16 Pearson correlations and the 16 Spearman correlations within each group. Associations were considered robust only when they were directionally consistent across parametric and non-parametric analyses and remained statistically significant after multiplicity correction.

### 2.6. Ethical Considerations

The study was conducted in accordance with the Declaration of Helsinki and applicable data-protection regulations. Personal data were anonymized before analysis, and files were stored in a secure database accessible only to the research team. Ethical approval was obtained from the Ethics Committee of Universidad Católica San Antonio (CE072508) on 25 July 2025. Informed consent was obtained from parents or legal guardians for all participating children.

## 3. Results

### 3.1. Sample Characteristics and Group Comparability

The analytic sample comprised 80 children, 40 with DLD and 40 TD peers. No missing values were detected. The total sample had a mean age of 8.08 years (SD = 0.31; range = 7.3–8.9). Levene tests were non-significant for all eight primary outcomes (*p* = 0.081–0.880), supporting the equal-variance *t* tests. Shapiro–Wilk tests indicated statistically significant departures from normality for 15 of the 16 within-group distributions. The only non-significant result was observed for verbal comprehension in the DLD group, *W* = 0.952, *p* = 0.086. Across the remaining distributions, Shapiro–Wilk statistics ranged from *W* = 0.855 to 0.921, with exact *p* values ranging from 0.000117 to 0.00824. These departures were consistent with the discrete and restricted nature of the scaled scores. Accordingly, Pearson correlations were supplemented with Spearman rank-order correlations. Equal-variance *t* tests were retained for the group comparisons because the two groups had equal sample sizes, no extreme outliers were detected, and Levene tests were non-significant for all eight outcomes.

Groups did not differ significantly in age, sex, maternal education, family structure, or school type. Socioeconomic status and the FSIQ eligibility criterion were invariant. Paternal university education was more frequent in the TD group, χ^2^(1) = 6.24, *p* = 0.012. Group-by-paternal-education interactions were non-significant for seven outcomes, with exact *p* values ranging from 0.168 to 0.902. For auditory integration, the interaction was statistically significant, F(1, 76) = 5.85, *p* = 0.018, indicating that the magnitude of the group difference varied according to paternal education (Table 1).

### 3.2. Group Differences in Auditory Psycholinguistic Abilities and Cognitive Outcomes

Children with DLD scored significantly lower than TD peers on all eight measures. Exact *p* values for the unadjusted comparisons ranged from 2.91 × 10^−18^ to 4.80 × 10^−8^. Standardized mean differences ranged from d = 1.35 for working memory to d = 2.55 for fluid reasoning, and all 95% confidence intervals excluded zero.

Sensitivity analyses were conducted to examine whether the between-group differences remained after accounting for paternal university education. For the seven outcomes with non-significant group-by-paternal-education interactions, adjusted group effects remained statistically significant, with exact *p* values ranging from 3.61 × 10^−17^ to 3.24 × 10^−8^. For auditory integration, the group-by-paternal-education interaction was statistically significant, F(1, 76) = 5.85, *p* = 0.018. Simple-effects analyses showed significantly lower auditory-integration scores in the DLD group both among children whose fathers did not have a university education, b = −2.82, SE = 0.45, t(76) = −6.26, *p* = 2.08 × 10^−8^, and among children whose fathers had a university education, b = −1.42, SE = 0.37, t(76) = −3.87, *p* = 0.000227. Thus, the DLD–TD difference remained statistically significant at both levels of paternal education, although it was larger in the non-university subgroup (Table 2).

### 3.3. Within-Group Associations Between Auditory Psycholinguistic Abilities and Cognitive Outcomes

Pearson analyses identified three nominally significant correlations and one additional near-threshold association. In the DLD group, auditory comprehension was positively correlated with fluid reasoning (r = 0.34, 95% CI [0.03, 0.59], *p* = 0.034). Auditory integration showed a negative association with processing speed that approached, but did not reach, the conventional significance threshold (r = −0.30, 95% CI [−0.56, 0.01], *p* = 0.056). In the TD group, auditory sequential memory was negatively correlated with working memory (r = −0.32, 95% CI [−0.57, −0.01], *p* = 0.045) and positively correlated with processing speed (r = 0.31, 95% CI [0.00, 0.57], *p* = 0.048). However, the corresponding Spearman coefficients were smaller and non-significant (|ρ| = 0.28–0.30, *p* = 0.059–0.085), and no association remained statistically significant after false-discovery-rate correction. Accordingly, the uncorrected Pearson coefficients should be interpreted as exploratory and not as robust evidence of stable within-group relationships (Table 3).

## 4. Discussion

The present study compared four auditory psycholinguistic abilities and four selected cognitive outcomes in children with developmental language disorder (DLD) and typically developing (TD) peers. It also examined whether the relationships between auditory psycholinguistic and cognitive performance were consistent within each developmental group. Three main findings emerged. First, children with DLD obtained substantially lower scores than TD peers across all auditory psycholinguistic and cognitive measures, with large effect sizes. Second, the between-group differences remained statistically significant after accounting for paternal education; for auditory integration, the difference was significant at both levels of paternal education, although its magnitude differed between strata. Third, the within-group associations were sparse and method-dependent: three Pearson correlations reached nominal significance before correction, but none of the Pearson or Spearman associations remained statistically significant after false-discovery-rate correction. Thus, H1 was supported, whereas H2 did not receive robust empirical support after sensitivity analysis and multiplicity correction. Taken together, these results suggest that DLD is associated with a broad but heterogeneous profile of language-related and cognitive vulnerabilities, whereas the relationships between specific auditory psycholinguistic abilities and cognitive outcomes appear less stable than the marked differences observed between groups.

The magnitude and consistency of the between-group differences are broadly consistent with previous research showing that children with DLD frequently experience difficulties not only in language processing but also in verbal short-term memory, working memory, processing efficiency, and some aspects of executive and nonverbal functioning (Archibald & Gathercole, 2006; Henry et al., 2012; Larson & Ellis Weismer, 2022; Miller et al., 2001; Vugs et al., 2013; Zapparrata et al., 2023). The present findings extend this evidence by showing that the DLD group performed more poorly across several higher-level auditory psycholinguistic operations, including auditory comprehension, auditory association, auditory integration, and auditory sequential memory, as well as across the selected cognitive measures representing verbal comprehension, working memory, fluid reasoning, and processing speed. This broad pattern is compatible with multifactorial accounts of DLD, according to which language difficulties may coexist with vulnerabilities in memory, processing efficiency, attentional control, and learning mechanisms rather than resulting from a single underlying deficit.

The adjusted analyses provide additional support for the robustness of the between-group differences. Paternal university education was more frequent in the TD group, raising the possibility that part of the observed performance gap could reflect differences in educational or home-learning opportunities. For seven outcomes, the group differences remained statistically significant after paternal education was included as a covariate. For auditory integration, the significant group-by-paternal-education interaction indicated that the magnitude of the DLD–TD difference varied across paternal-education strata; nevertheless, simple-effects analyses showed significantly lower scores in the DLD group at both levels of paternal education. This reduces concern that the principal findings were explained solely by this sociodemographic imbalance. Nevertheless, residual confounding cannot be completely excluded. Paternal education was dichotomized, and other potentially relevant characteristics of the home environment, such as family literacy practices, parental language input, access to educational resources, and the intensity of previous intervention, were not measured. Therefore, the adjusted findings should be interpreted as evidence of robustness to one measured sociodemographic difference rather than as proof that environmental factors played no role.

The cognitive results also require careful interpretation because the study used selected WISC-V age-scaled subtest scores rather than complete composite indices. These scores provide clinically meaningful information about performance on specific tasks, but they should not be considered equivalent to full indices of verbal comprehension, working memory, fluid reasoning, or processing speed. Accordingly, the present findings do not establish generalized impairments in global intellectual functioning. Nor do they imply that every child with DLD shows the same pattern of cognitive difficulty. Rather, they indicate that, at the group level, children with DLD obtained lower scores on the selected tasks used to represent these domains. This distinction is important because DLD is characterized by considerable interindividual variability, and group means may obscure clinically relevant differences in strengths, weaknesses, and compensatory resources.

A central contribution of the study lies in its distinction between between-group differences and within-group covariation. When two groups differ substantially in their mean scores across several domains, analyses conducted on the combined sample may produce apparently strong cross-domain associations that are driven largely by diagnostic group membership. Such associations can be misinterpreted as evidence that one domain directly explains another. By calculating correlations separately within the DLD and TD groups, the present study reduced this source of ecological or compositional bias. The resulting within-group pattern was notably weaker than the group contrasts, indicating that the coexistence of lower auditory psycholinguistic and cognitive scores in DLD does not necessarily imply that these measures vary together in a simple or uniform way among children within the disorder.

The limited stability of the within-group associations is therefore an important finding. In the DLD group, auditory comprehension showed a nominal positive Pearson correlation with fluid reasoning. One possible interpretation is that, for some children with DLD, performance on reasoning tasks may depend partly on language-mediated strategies, including the verbal encoding of relationships, maintenance of task instructions, and the use of lexical labels to organize abstract information. Under this interpretation, stronger auditory comprehension could provide linguistic scaffolding that supports relational reasoning. This association might be more observable in children with DLD because language processing is less automatic and may therefore contribute more strongly to individual differences in task performance. In TD children, by contrast, more efficient language processing or greater availability of alternative nonverbal strategies could reduce the observable association between these domains.

However, this explanation must remain tentative. The correlation was not statistically significant in the Spearman sensitivity analysis and did not survive correction for multiple testing. The result may therefore reflect sampling variability, shared task demands, or the influence of unmeasured factors such as attention, motivation, comprehension of instructions, or executive control. In addition, fluid reasoning was represented by a selected WISC-V subtest rather than by a comprehensive latent assessment of reasoning ability. Consequently, the observed coefficient should be treated as hypothesis-generating rather than as evidence of a DLD-specific mechanism.

A similarly cautious interpretation is required for the negative association between auditory integration and processing speed observed in the DLD group. From a theoretical perspective, one might speculate that some children achieved better auditory-integration scores by adopting a more deliberate and accuracy-oriented response style, whereas stronger performance on timed processing-speed tasks may reflect a different balance between speed and accuracy. It is also possible that children with less efficient automatic processing rely more heavily on effortful linguistic reconstruction or contextual inference, producing an apparent inverse association between integration performance and speeded output. Statistical suppression or task-specific variance could provide alternative explanations.

Nevertheless, none of these interpretations can be established from the present data. The coefficient did not reach the conventional significance threshold in the Pearson analysis, was non-significant in the Spearman analysis, and did not survive false-discovery-rate correction. Furthermore, the processing-speed measure involved rapid visual scanning, coding, and graphomotor output rather than a pure measure of auditory or linguistic processing speed. The observed direction may therefore reflect measurement-specific demands rather than a meaningful inverse relationship between auditory integration and general processing efficiency. Future studies should examine this possibility using direct reaction-time tasks, separate indices of speed and accuracy, and measures that distinguish auditory processing speed from visual-motor speed.

The unexpected negative association between auditory sequential memory and working memory in the TD group also warrants restraint. Conceptually, a positive relationship would ordinarily be expected because both constructs involve the short-term retention and ordering of information. The negative direction is therefore unlikely to support a straightforward theoretical account. Restricted score variability in the TD group, task-specific strategies, ceiling effects, measurement error, or chance fluctuation in a relatively small sample may have contributed. Because the relationship was not statistically significant in the Spearman analysis and did not survive multiplicity correction, it should not be interpreted as evidence that stronger auditory sequential memory is associated with weaker working memory in typical development.

The positive nominal relationship between auditory sequential memory and processing speed in the TD group should be interpreted in the same way. It is theoretically plausible that more efficient short-term ordering of auditory information may accompany broader processing efficiency. However, the association did not remain statistically significant in the non-parametric analysis and did not survive false-discovery-rate correction. The finding therefore provides, at most, preliminary evidence that should be examined in larger and more precisely measured samples.

More broadly, the contrast between large group differences and weak within-group correlations illustrates an important distinction between group-level impairment and individual-level mechanisms. Two diagnostic groups may differ substantially across multiple measures without variation in one domain explaining variation in another within each group. In the present study, the strong group contrasts did not translate into stable evidence that auditory psycholinguistic abilities were closely coupled with cognitive performance among children with DLD or among TD peers. This pattern argues against a simple model in which the assessed auditory psycholinguistic abilities constitute a uniform explanation for the broader cognitive profile associated with DLD.

Instead, the results are more compatible with a multifactorial and heterogeneous interpretation. Language-related and cognitive difficulties may arise from partially overlapping but non-identical influences, including the quality of linguistic representations, verbal memory capacity, processing efficiency, sustained attention, executive control, learning mechanisms, and environmental experience. Different combinations of these factors may produce similar observable language difficulties across children. Under such a model, auditory psycholinguistic and cognitive vulnerabilities may coexist, but their relationships may vary according to the child’s developmental history, severity of language impairment, compensatory strategies, educational experience, and comorbid symptoms.

The lack of robust within-group associations also argues against interpreting the auditory psycholinguistic measures as a single explanatory mechanism for the cognitive differences between groups. Strong between-group differences across two sets of measures do not imply that variation in one domain accounts for variation in the other. The weak and imprecise within-group associations observed in the present study do not provide a sufficiently robust basis for a unitary explanatory account. This does not demonstrate that auditory psycholinguistic processes are irrelevant to cognitive functioning; rather, it suggests that their relationships with the selected cognitive measures are likely to be selective, heterogeneous, and influenced by additional developmental and contextual factors.

From a clinical perspective, the findings reinforce the value of comprehensive and individualized assessment. The coexistence of substantial group differences across auditory-language and cognitive tasks does not justify assuming that all children with DLD present the same underlying deficit. Nor does it support using a single auditory measure as a proxy for broader cognitive functioning. Assessment should therefore characterize auditory comprehension, semantic association, auditory integration, auditory sequential memory, working memory, processing speed, and reasoning separately. Where clinically relevant, attention, executive functioning, literacy, academic attainment, and everyday communicative functioning should also be assessed.

A multidimensional approach may help clinicians distinguish primary areas of difficulty from secondary consequences and identify compensatory strengths. For example, one child may show marked auditory sequential-memory difficulties but relatively preserved processing speed, whereas another may present broader inefficiencies across several domains. These profiles may require different forms of support even when both children meet the criteria for DLD. Profile-based assessment is therefore likely to be more clinically informative than assuming a single cognitive-linguistic pathway shared by all children with the disorder.

The findings also have implications for intervention planning, although the present design does not provide evidence of treatment efficacy. For children who show reduced processing efficiency, clinicians and educators may consider reducing verbal load, presenting information in shorter units, allowing additional response time, repeating or visually supporting instructions, and checking comprehension explicitly. Children with weaknesses in sequential memory may benefit from external memory supports, structured rehearsal, chunking, and the gradual increase in verbal complexity. Difficulties in auditory comprehension or association may require explicit vocabulary teaching, semantic organization, contextual support, and repeated opportunities to integrate verbal information.

However, the absence of robust cross-domain associations cautions against assuming that improvement in auditory psycholinguistic skills will automatically produce generalized gains in working memory, reasoning, or processing speed. Intervention targets should be selected according to each child’s demonstrated needs, and treatment outcomes should be evaluated within the specific domains addressed. Claims regarding transfer from language-focused intervention to broader cognitive functioning require direct testing in randomized or longitudinal intervention studies.

Several limitations should be considered when interpreting the findings. First, the sample size was sufficient to detect the large between-group differences observed but provided limited sensitivity for small-to-moderate within-group correlations. With 40 participants per group, only relatively large associations could be detected with conventional statistical power. Consequently, non-significant correlations may reflect imprecision rather than the complete absence of a relationship. Confidence intervals around several coefficients remained compatible with effects in different directions, underscoring the need for larger samples.

Second, the cross-sectional design precludes conclusions regarding causality, temporal ordering, or developmental change. The study cannot determine whether auditory psycholinguistic difficulties precede cognitive differences, result partly from them, or develop reciprocally over time. Longitudinal studies are needed to establish whether early auditory psycholinguistic performance predicts later cognitive or academic outcomes and whether changes in one domain are associated with subsequent changes in another.

Third, DLD status was based on prior clinical or educational documentation and parent confirmation rather than on a newly administered comprehensive diagnostic battery. Although this approach reflects real-world identification practices, the original diagnostic procedures may have differed across participants. This variation may have increased heterogeneity within the DLD group. Future studies should include standardized characterization of receptive and expressive language severity, functional impact, intervention history, and relevant comorbid symptoms.

Fourth, complete examiner blinding could not be guaranteed. Although the examiner did not participate in the original diagnostic classification, language difficulties or information contained in the participants’ records may have made group membership apparent. This could have introduced expectancy effects during administration or scoring, although the use of standardized procedures may have reduced this risk.

Fifth, the sample was relatively homogeneous in socioeconomic level, and recruitment from a limited number of educational centres and specialized services may restrict generalizability. The dichotomization of parental education may also have obscured more gradual socioeconomic influences. Future research should include participants from more diverse linguistic, cultural, educational, and socioeconomic backgrounds and should assess the home language and literacy environment in greater detail.

Sixth, the FSIQ eligibility threshold restricted the lower range of intellectual functioning. Although this criterion was used to delimit the analytic sample and reduce the likelihood that the observed group differences were attributable to global intellectual disability, it may also have excluded children with clinically significant language difficulties and lower general cognitive scores. Accordingly, the findings apply most directly to children with DLD whose documented intellectual functioning fell within the specified range. This threshold should not be interpreted as a universal diagnostic requirement for DLD, because current consensus recommendations do not consider low nonverbal ability alone sufficient to exclude the diagnosis.

Seventh, the cognitive outcomes were based on selected WISC-V age-scaled subtest scores rather than complete composite indices. These measures may contain variance related to comprehension of instructions, visual-motor demands, attention, response style, and test-taking strategies. The findings should therefore be understood as task-specific indicators rather than comprehensive estimates of the cognitive domains represented.

Eighth, the study did not include direct measures of executive functioning, sustained attention, inhibition, cognitive flexibility, lower-level auditory perception, or reaction time. These unmeasured processes may contribute to both auditory psycholinguistic and cognitive performance and could help explain why some children with DLD present broader difficulties than others. Their omission limits the ability to distinguish language-specific from domain-general influences.

Finally, the number of exploratory correlations increased the probability of chance findings. After false-discovery-rate correction, no within-group association remained statistically significant. The nominal Pearson coefficients should therefore be regarded as preliminary signals requiring independent replication rather than as established effects. The use of Spearman correlations and multiplicity correction increased analytical robustness, but it also highlighted the uncertainty surrounding the uncorrected results.

Future research should recruit larger and more diverse samples, employ longitudinal designs, and include multiple indicators of each construct to reduce task-specific measurement error. Latent-variable approaches may be particularly useful for distinguishing shared cognitive-linguistic variance from variance attributable to individual tests. Studies should also incorporate direct measures of executive control, attention, auditory temporal processing, and reaction time, as well as detailed information about language severity, educational support, intervention history, and comorbid symptoms. Such designs could determine whether distinct subgroups of children with DLD show different patterns of association between auditory psycholinguistic and cognitive performance.

## 5. Conclusions

In conclusion, the present study demonstrates marked differences between children with DLD and TD peers across the assessed auditory psycholinguistic and cognitive measures. Its principal contribution is to show that these broad group differences were not accompanied by equally strong or stable associations within each developmental group. The findings therefore challenge a simple account in which auditory psycholinguistic weakness uniformly explains the cognitive profile associated with DLD. Instead, they support a multifactorial and heterogeneous interpretation in which language-related and cognitive vulnerabilities may coexist, interact selectively, and vary across children. This perspective favors multidimensional assessment and individualized intervention rather than the assumption of a single underlying deficit or a uniform pathway from auditory-language performance to broader cognition.

## Figures and Tables

**Table 1:** Sociodemographic characteristics and group comparisons.

Variable	DLD (n = 40)	TD (n = 40)	Test	*p*
Age, years	8.08 (0.35)	8.08 (0.27)	t(78) = 0.04	0.972
Sex, boys	22 (55.0%)	21 (52.5%)	χ^2^(1) = 0.05	0.823
Father’s education, university	18 (45.0%)	29 (72.5%)	χ^2^(1) = 6.24	0.012
Mother’s education, university	28 (70.0%)	27 (67.5%)	χ^2^(1) = 0.06	0.809
Family structure, nuclear	37 (92.5%)	37 (92.5%)	χ^2^(1) = 0.00	1.000
School type, public	31 (77.5%)	24 (60.0%)	χ^2^(1) = 2.85	0.091
Socioeconomic status, medium	40 (100%)	40 (100%)	Not tested	—
FSIQ eligibility criterion, ≥85	40 (100%)	40 (100%)	Not tested	—

Note. Percentages are within the group. DLD = developmental language disorder; TD = typically developing. Socioeconomic status and FSIQ eligibility showed no variability and were not tested inferentially.

**Table 2:** Unadjusted and paternal-education-adjusted group differences in auditory psycholinguistic abilities and cognitive outcomes.

Measure	DLD M (SD)	TD M (SD)	t(78)	Unadjusted *p*	95% CI Mean Difference	Cohen’s d [95% CI]	Adjusted Group F(1, 77)	Adjusted *p*
Auditory comprehension	6.03 (1.05)	8.40 (0.90)	−10.86	2.85 × 10^−17^	[−2.81, −1.94]	2.43 [1.90, 3.17]	101.98	9.38 × 10^−16^
Auditory association	6.63 (1.10)	8.15 (1.03)	−6.40	1.05 × 10^−8^	[−2.00, −1.05]	1.43 [0.95, 2.02]	40.41	1.33 × 10^−8^
Auditory integration	6.23 (1.25)	8.28 (1.26)	−7.30	2.09 × 10^−10^	[−2.61, −1.49]	1.63 [1.16, 2.28]	- ^1^	- ^1^
Sequential memory	5.85 (1.29)	8.23 (0.92)	−9.47	1.31 × 10^−14^	[−2.87, −1.88]	2.12 [1.67, 2.78]	80.49	1.36 × 10^−13^
Verbal comprehension	6.63 (1.39)	9.18 (1.03)	−9.31	2.76 × 10^−14^	[−3.10, −2.00]	2.08 [1.59, 2.71]	79.12	1.91 × 10^−13^
Working memory	6.90 (1.43)	8.63 (1.10)	−6.05	4.80 × 10^−8^	[−2.29, −1.16]	1.35 [0.94, 1.83]	37.80	3.24 × 10^−8^
Fluid reasoning	6.93 (1.02)	9.45 (0.96)	−11.39	2.91 × 10^−18^	[−2.97, −2.08]	2.55 [1.95, 3.33]	117.64	3.61 × 10^−17^
Processing speed	6.78 (1.14)	8.43 (0.81)	−7.44	1.14 × 10^−10^	[−2.09, −1.21]	1.66 [1.18, 2.30]	47.41	1.37 × 10^−9^

Note. Mean differences were computed as DLD minus TD. Cohen’s d is reported in the TD-minus-DLD direction, so positive values indicate higher performance in the TD group. Adjusted group effects were obtained from linear models including group and paternal university education. ^1^ For auditory integration, the group-by-paternal-education interaction was significant, F(1, 76) = 5.85, *p* = 0.018; therefore, a single adjusted group effect from the reduced model is not reported. Simple-effects analyses showed significant DLD–TD differences both for non-university paternal education, b = −2.82, SE = 0.45, t(76) = −6.26, *p* = 2.08 × 10^−8^, and university paternal education, b = −1.42, SE = 0.37, t(76) = −3.87, *p* = 0.000227.

**Table 3:** Pearson and Spearman correlations between auditory psycholinguistic abilities and cognitive outcomes by group.

Group	Auditory Measure	Cognitive Outcome	Pearson r [95% CI]	Pearson *p*	Pearson FDR *p*	Spearman ρ [95% CI]	Spearman *p*	Spearman FDR *p*
DLD	Auditory comprehension	Verbal comprehension	−0.12 [−0.41, 0.20]	0.474	0.972	−0.07 [−0.37, 0.25]	0.680	0.943
	Auditory comprehension	Working memory	−0.08 [−0.39, 0.23]	0.607	0.972	−0.07 [−0.40, 0.25]	0.653	0.943
	Auditory comprehension	Fluid reasoning	0.34 [0.03, 0.59]	0.034	0.448	0.28 [−0.09, 0.59]	0.085	0.679
	Auditory comprehension	Processing speed	0.00 [−0.31, 0.32]	0.977	0.993	0.05 [−0.26, 0.36]	0.766	0.943
	Auditory association	Verbal comprehension	0.06 [−0.26, 0.36]	0.729	0.972	0.04 [−0.28, 0.36]	0.813	0.943
	Auditory association	Working memory	0.02 [−0.29, 0.33]	0.881	0.993	−0.01 [−0.35, 0.35]	0.969	0.969
	Auditory association	Fluid reasoning	−0.09 [−0.39, 0.22]	0.565	0.972	−0.03 [−0.33, 0.26]	0.837	0.943
	Auditory association	Processing speed	−0.15 [−0.44, 0.17]	0.355	0.972	−0.11 [−0.42, 0.22]	0.487	0.943
	Auditory integration	Verbal comprehension	0.08 [−0.24, 0.38]	0.627	0.972	0.06 [−0.24, 0.37]	0.701	0.943
	Auditory integration	Working memory	0.00 [−0.31, 0.31]	0.993	0.993	−0.02 [−0.32, 0.27]	0.884	0.943
	Auditory integration	Fluid reasoning	−0.15 [−0.44, 0.17]	0.366	0.972	−0.16 [−0.47, 0.16]	0.314	0.943
	Auditory integration	Processing speed	−0.30 [−0.56, 0.01]	0.056	0.448	−0.29 [−0.58, 0.04]	0.074	0.679
	Sequential memory	Verbal comprehension	−0.06 [−0.37, 0.26]	0.710	0.972	−0.06 [−0.39, 0.25]	0.702	0.943
	Sequential memory	Working memory	−0.04 [−0.34, 0.28]	0.825	0.993	−0.07 [−0.37, 0.26]	0.689	0.943
	Sequential memory	Fluid reasoning	−0.07 [−0.37, 0.25]	0.681	0.972	−0.10 [−0.44, 0.26]	0.543	0.943
	Sequential memory	Processing speed	0.10 [−0.22, 0.40]	0.547	0.972	0.03 [−0.30, 0.36]	0.866	0.943
TD	Auditory comprehension	Verbal comprehension	−0.05 [−0.36, 0.27]	0.761	0.989	−0.08 [−0.40, 0.24]	0.613	0.775
	Auditory comprehension	Working memory	−0.13 [−0.42, 0.19]	0.427	0.723	−0.12 [−0.46, 0.23]	0.458	0.775
	Auditory comprehension	Fluid reasoning	0.17 [−0.15, 0.46]	0.288	0.670	0.13 [−0.19, 0.44]	0.418	0.775
	Auditory comprehension	Processing speed	0.01 [−0.31, 0.32]	0.966	0.989	0.07 [−0.23, 0.38]	0.678	0.775
	Auditory association	Verbal comprehension	0.22 [−0.10, 0.49]	0.181	0.670	0.21 [−0.11, 0.50]	0.196	0.775
	Auditory association	Working memory	−0.20 [−0.48, 0.12]	0.220	0.670	−0.15 [−0.42, 0.13]	0.368	0.775
	Auditory association	Fluid reasoning	−0.12 [−0.42, 0.20]	0.452	0.723	−0.12 [−0.42, 0.22]	0.474	0.775
	Auditory association	Processing speed	−0.17 [−0.46, 0.15]	0.293	0.670	−0.18 [−0.46, 0.13]	0.275	0.775
	Auditory integration	Verbal comprehension	−0.21 [−0.49, 0.10]	0.183	0.670	−0.26 [−0.54, 0.06]	0.103	0.551
	Auditory integration	Working memory	0.00 [−0.31, 0.31]	0.989	0.989	−0.04 [−0.38, 0.30]	0.819	0.873
	Auditory integration	Fluid reasoning	−0.08 [−0.39, 0.23]	0.607	0.883	−0.07 [−0.37, 0.25]	0.668	0.775
	Auditory integration	Processing speed	−0.02 [−0.33, 0.30]	0.918	0.989	0.03 [−0.27, 0.34]	0.873	0.873
	Sequential memory	Verbal comprehension	−0.12 [−0.42, 0.20]	0.449	0.723	−0.10 [−0.38, 0.19]	0.551	0.775
	Sequential memory	Working memory	−0.32 [−0.57, −0.01]	0.045	0.384	−0.28 [−0.57, 0.06]	0.079	0.551
	Sequential memory	Fluid reasoning	0.03 [−0.29, 0.34]	0.866	0.989	0.08 [−0.22, 0.40]	0.640	0.775
	Sequential memory	Processing speed	0.31 [0.00, 0.57]	0.048	0.384	0.30 [−0.01, 0.57]	0.059	0.551

Note. Pearson confidence intervals were calculated using Fisher’s *z* transformation. Spearman confidence intervals were obtained by bootstrap resampling. Benjamini–Hochberg false-discovery-rate correction was applied separately to the 16 Pearson correlations and the 16 Spearman correlations within each group. Three Pearson correlations reached nominal significance before correction: auditory comprehension with fluid reasoning in the DLD group and sequential memory with working memory and processing speed in the TD group. The association between auditory integration and processing speed in the DLD group approached but did not reach the conventional significance threshold. No Pearson or Spearman correlation remained statistically significant after FDR correction. DLD = developmental language disorder; TD = typically developing.

## Data Availability

The data presented in this study are available on request from the corresponding author. The data are not publicly available due to specific ethical and privacy considerations.
